# Rational design and investigation of nonlinear optical response properties of pyrrolopyrrole aza-BODIPY-based novel push–pull chromophores†

**DOI:** 10.1039/d4ra02861a

**Published:** 2024-05-16

**Authors:** Naga Pranava Sree Kothoori, Pandiyan Sivasakthi, Mallesham Baithy, Ramprasad Misra, Pralok K. Samanta

**Affiliations:** a Department of Chemistry, School of Science, Gandhi Institute of Technology and Management (GITAM) Hyderabad-502329 India pralokkumar.samanta@hyderabad.bits-pilani.ac.in; b Institute for Biology, Experimental Biophysics, Humboldt-Universität zu Berlin Berlin-10115 Germany ramprasad.misra@hu-berlin.de ramprasadmisra@gmail.com; c Department of Chemistry, Birla Institute of Technology and Science Pilani (BITS Pilani), Hyderabad Campus Hyderabad-500078 India

## Abstract

Intramolecular charge transfer (ICT)-based chromophores are highly sought after for designing near-infrared (NIR) absorbing and emitting dyes as well as for designing materials for nonlinear optical (NLO) applications. The properties of these ‘push–pull’ molecules can easily be modified by varying the electronic donor (D) and acceptor (A) groups as well as the π-conjugation linker. This study presents a methodical approach and employs quantum chemical analysis to explore the relationship between the structural features, electro-optical properties, and the NLO characteristics of molecules with D–π–A framework. The one- and two-photon absorption (2PA), linear polarizability (*α*), and first hyperpolarizability (*β*) of some novel chromophores, consisting of a dimeric aza-Boron Dipyrromethene (aza-BODIPY) analogue, called, pyrrolopyrrole aza-BODIPY (PPAB), serving as the acceptor, have been investigated. The electronic donors used in this study are triphenylamine (TPA) and diphenylamine (DPA), and they are conjugated to the acceptor *via* thienyl or phenylene π-linkers. Additionally, the Hyper–Rayleigh Scattering (*β*_HRS_), which enables direct estimation of the second-order NLO properties, is calculated for the studied chromophores with 1064 nm excitation in acetonitrile. The *β* value shows a significant increase with increasing solvent polarity, indicating that the ICT plays a crucial role in shaping the NLO response of the studied molecules. The enhancement of the 2PA cross-section of the investigated molecules can also be achieved by modulating the combinations of donors and linkers. The results of our study indicate that the novel D–π–A molecules designed in this work demonstrate considerably higher hyperpolarizability values than the standard *p*-nitroaniline, making them promising candidates for future NLO applications.

## Abstract 2

Intramolecular charge transfer plays crucial role in shaping linear and nonlinear optical response properties of novel pyrrolopyrrole aza-BODIPY-based push–pull chromophores.

## Graphical abstract

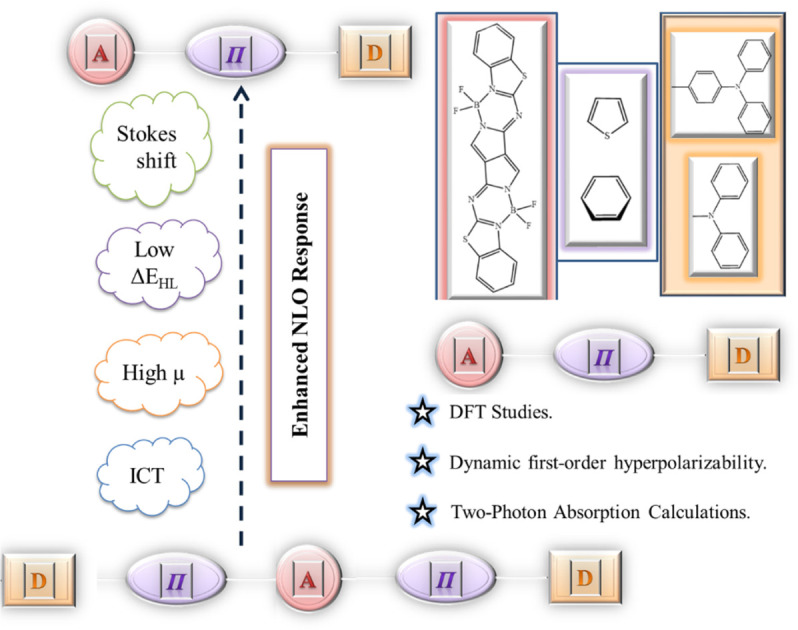

## 1. Introduction

Nonlinear optical (NLO) materials are crucial for the development of contemporary technologies, including optical communications, signal processing, and data storage.^1–4^ Materials with optical limiting (OL) properties have the ability to effectively absorb significant amounts of high-energy lasers, thereby lowering the output energy. This is a key part of protecting eyes and optical systems from damage caused by lasers.^5,6^ The demand for designing new NLO materials has significantly increased in the field of optoelectronics and photonics in recent years. The unique photo-physical properties exhibited by NLO materials under intense laser irradiation account for their extensive range of practical uses. Organic compounds are highly sought after for designing novel NLO materials owing to their capability to achieve rapid response rates, increased photo-electric quantum efficiency, and low dielectric constants.^7^ Organic molecules also possess greater design freedom in comparison to inorganic substances, rendering them more economically efficient compared to their inorganic counterparts.^8^ In order for a molecule to demonstrate NLO characteristics, it must have a significant initial hyperpolarizability and non-centrosymmetric geometry.^9,10^ Organic chromophores that display strong absorption and emission in the near-infrared (NIR) region are useful for several technological applications, including, solar cells, heat absorbers and NIR-emitting diodes.^11,12^

**Fig. 1:**
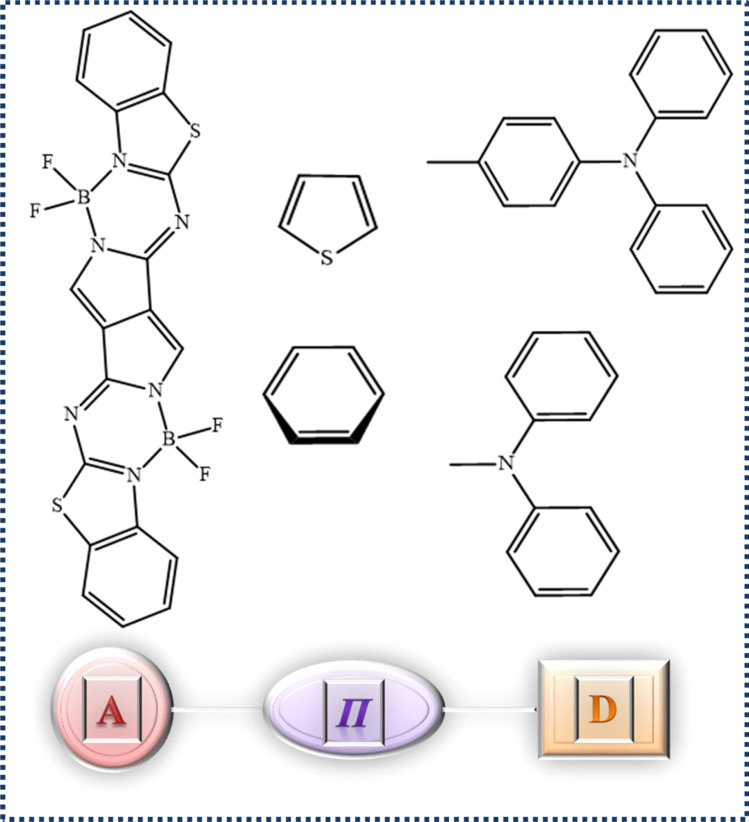
The 2-D representation of the acceptor, linker, and donor components.

BODIPY molecules exhibit notable absorption properties and demonstrate highly efficient fluorescence emission in the NIR range.^13,14^ The absorption and emission characteristics of these molecules can be readily altered by changing the substitution pattern of the BODIPY framework, leading to an increase in their fluorescence in the NIR spectrum.^15^ These dyes are well-known for their remarkable resistance to heat and light-induced chemical reactions and their capacity to produce fluorescent sensors, and hence aza-BODIPY dyes are very desirable organic materials for a wide range of applications.^16^ Pyrrolopyrrole aza-BODIPY (PPAB), as illustrated in Fig. 1, exhibits a broad absorption spectrum in the visible and near-infrared regions due to its extensive conjugate structure.^17–19^ Dyes with NIR absorption and emission properties are preferred in biological assays and screening procedures due to reduced interference from auto fluorescence and enhanced capacity to penetrate deeper into the sample.^20–22^ Lately, intramolecular charge transfer (ICT) based molecules with electron-donating and electron-accepting components, connected either together or through π-linkers, have become popular for designing chromophores for NLO materials. Incorporating suitable electronic donor (D) and acceptor (A) components on opposite ends of the π-linker, such as in molecules with D–A, D–π–A, D–A–D, and A–D–A structures, can lead to a noteworthy second-order NLO response.^23–26^

**Fig. 2:**
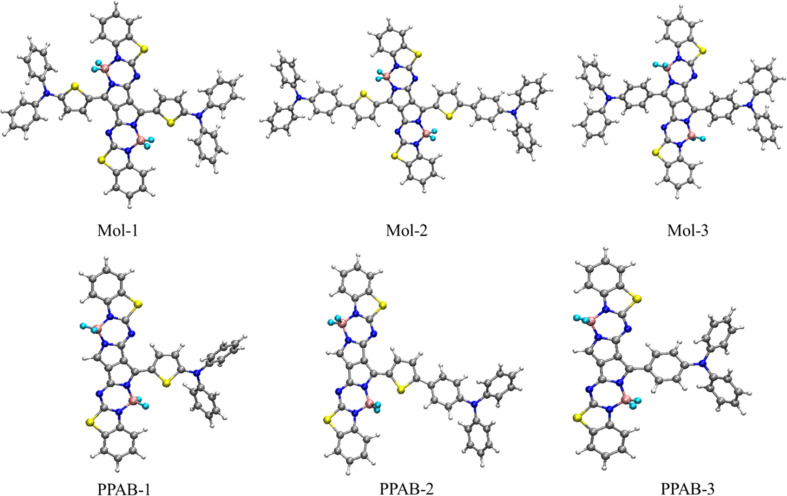
Design strategy of novel PPAB chromophores for NLO applications: upper panel shows the optimized geometries of D–π–A–π–D series of molecules (1–3), used earlier for designing NIR-emitting dyes [ref. 27]. As NLO response of these molecules are quite low, presumably due to their centrosymmetric nature, using the same donor and acceptor groups we have designed D–π–A chromophores PPAB (1–3), whose optimized geometries are shown in the lower panel.

Recently, Wang *et al.* reported a series of molecules with a donor–acceptor–donor (D–π–A–π–D) framework, with the acceptor component consists of pyrrolopyrrole aza-BODIPYs, while the donors are TPA or DPA, connected by a thienyl or phenylene linker.^27^ They studied the singlet emission properties of these molecules, which were observed in the NIR region. In this study, we investigated the absorption and NLO response properties of the aforesaid molecules with (D–π–A–π–D) structure, which led us to design molecules with D–π–A architecture for obtaining chromophores with higher NLO responses. The PPAB core is present in all three compounds, as shown in Fig. 2, and these molecules exhibit a high level of resistance to light-induced degradation, which is a phenomenon usually exhibited by inorganic frameworks containing NLO molecules.^28^ A π-conjugated linker connects electron-donating and electron-withdrawing groups in a molecule. In our study, the π-conjugation bridge (a thienyl or phenylene linker) increases ICT, which leads to an enhancement in the NLO characteristics of the molecules.^29–31^ Varying the nature of the donor groups and the π-conjugated bridge by means of chemical design is the most common strategy for tuning the first hyperpolarizability (*β*_tot_) and by structural modification of the donor and π-linkers the dipolar molecules studied outperform current expectations of NLO materials.^32^ We also studied the second-order NLO properties of the PPAB molecules, which form the basis for the design of the NLO switching. Images produced using two-photon absorption (2PA) photoluminescence is reported to be more advantageous than one-photon induced photoluminescence due to its ability to provide three-dimensional resolution.^33^ Additionally, when excitation is conducted in the NIR range, it enables greater penetration depth.^34,35^ The PPAB-cored molecules exhibit high efficiency in the NIR region, with 2PA cross sections of approximately 3000 GM within the telecommunication window at wavelengths ranging from 1500 to 1700 nm.^36^ The current study may assist in understanding the ICT process in PPAB based dipolar PPAB chromophores and designing novel materials based on it.

## 2. Computational details

The ground state geometries were optimized using Density Functional Theory (DFT) with the range-separated parameter (*ω*) tuned ω*B97XD functional and 6-31G* basis set. The optimized *ω* values were estimated by minimizing *J*^2^ as follows:^37,38^1
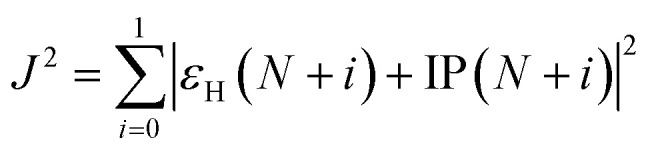
where IP and *ε*_H_ are the ionization potential and HOMO energy of a given molecule, respectively, and *N* is the total number of electrons in the molecule. Absorption spectra are obtained by the implementation of time-dependent DFT (TDDFT) methods. We have tested the calculated results with different DFT functionals (*viz.*, B3LYP, PBE1PBE, CAM-B3LYP, ωB97XD, and ω*B97XD) and found tuned ω*B97XD functional provide results closed to experimentally reported data. The computational calculations were performed using the Gaussian 16 quantum chemical software.^39^ The absorption and NLO response of the molecules are analyzed in three solvents of different polarities: acetonitrile, chloroform, and toluene. Acetonitrile, a highly polar solvent, is chosen to investigate the dynamic nonlinear optical characteristics of the molecules under study. Gaussian 16 utilizes the integral equation formalism of the polarizable continuum model (IEF-PCM) as its solvation model. It has been used to mimic the presence of solvent by creating a dielectric medium.^40^ Several properties of the molecules such as the dipole moment, average polarizability, and first hyperpolarizability of the chromophores were studied after the geometry optimization of the molecules. The dipole moment of the molecules is calculated using eqn (2).

2 *μ* = (*μ*_*x*_^2^ + *μ*_*y*_^2^ + *μ*_*z*_^2^)^1/2^

In the above equation, *x*, *y* and *z* denote the direction, and the *μ*_*x*_, *μ*_*y*_, *μ*_*z*_ are the dipole moment components of the molecule.^41^ The frontier molecular orbital (FMO) images, including the highest occupied molecular orbital (HOMO) and the lowest unoccupied molecular orbital (LUMO) are analyzed to estimate the band gap in the molecules and also to obtain global reactivity indices. Visual molecular dynamics (VMD) software was used to analyze and visualize the FMOs.^42^ The percentage of charge transfer (CT) and local excitation (LE) between the lowest excited singlet and triplet states is calculated *via* inter fragment charge transfer method using Multiwfn code.^43^ Electron density images of PPAB molecules in acetonitrile solvent are produced with an isosurface value of 0.001, as depicted in Fig. S3.† The 2PA calculations were done with B3LYP functional and 6-31G(d) basis set as implemented in DALTON software using ω*B97XD optimized coordinates.^44^

## 3. Results and discussion

### 3.1. Comparison between push–pull dipolar and quadrupolar chromophores

As molecules absorbing or emitting in the NIR region are highly intriguing, we examined the absorption and NLO response of Mol (1–3) with D–π–A–π–D architecture (Fig. 2).^27^ The ground state geometry of these molecules was optimized, followed by the computation of their first hyperpolarizability. Before computing their NLO response properties, the one-photon absorption of these molecules was studied using time-dependent density functional theory (TD-DFT) to find out the best basis set/functional combination. Several functionals including B3LYP, PBE1PBE, CAM-B3LYP, ωB97XD, and ω*B97XD, with a 6-31G(d) basis set were used to study the absorption of the aforesaid molecules (Tables 1 and S1†). Our analysis revealed that the tuned functional exhibited minimal divergence from the experimental absorption values. As the NLO properties of quadrupolar molecules with the D–π–A–π–D framework was not very satisfying (see Tables S2 and S3†), presumably due to their Centro-symmetric nature, basic molecular structure modifications to D–π–A were made, which helped us greatly enhance the NLO response of the molecules.

**Table 1:** Calculation of one-photon absorption energy (*λ*_abs_) with different DFT functionals. The experimental values are obtained from [ref. 27]. All values are provided in nm

Molecules	^27^Expt.	ω*B97XD	ωB97XD	CAM-B3LYP	B3LYP	PBE1PBE
Mol-1	816	752.19 (*ω* = 0.0938)	690.15	701.15	792.21	769.86
Mol-2	797	727.47 (*ω* = 0.0901)	654.85	671.09	855.07	808.79
Mol-3	728	692.47 (*ω* = 0.0961)	611.63	626.46	776.61	743.25

By taking the D–π–A–π–D structures as the basis, the electronic structure calculations for the D–π–A molecules were performed using (ω*B97XD) functional and 6-31G(d) basis set and the ground state optimized geometries of the molecules PPAB (1–3) are shown in Fig. 2. The absence of imaginary frequencies in the vibrational frequency analysis indicates that the optimised geometries have reached at least one of their local minima. In the present work, we theoretically studied the molecules with D–π–A structures in which the PPAB acts as an electron acceptor, TPA and DPA act as electron donors and the thienyl or phenylene molecules can be π-conjugated linkers.

### 3.2. Charge transfer and locally excited transitions

The ICT is an important factor that affects the NLO properties of organic molecules. Among the PPAB chromophores, PPAB-2 show a higher percentage of CT character which results in higher *β*_tot_ values, owing to the thienyl group which is a typical electron-rich substituent. Therefore, the introduction of thienyl as a bridge between the A–D is beneficial to enhance the ICT which provides a large transition dipole moment, which can strongly affect the polarizability of the molecule. The tendency to engage in ICT from TPA/DPA to PPAB moieties, facilitated by suitable π-linkers, is a significant factor in understanding the structure–function relationship for the design of NLO materials. It is known that a strong electron ‘push–pull’ effect has a positive influence on the NLO performance of molecules, and a high electron-donating capacity in a molecule with D–π–A architecture benefits the ICT. Consequently, the electron donating group of PPAB-2 has a greater capacity to donate electrons compared to PPAB-1. This results in a higher ICT character of the excited state of this molecule. The higher ICT character is achieved by replacing the DPA (donor) with the TPA (another donor) in the structure.

### 3.3. Solvent effect on one-photon absorption

The calculation of the one photon absorption energies, the oscillator strength (*f*) and the fluorescence emission of PPAB (1–3) molecules is performed using the TD-DFT method. As the change in solvent polarity is likely to result in a substantial alteration of the optical characteristics of ICT-based molecules,^45^ we go beyond gas-phase calculations to investigate the impact of a continuum dielectric on the electronic structure and properties, such as the dipole moment (*μ*), average polarizability (*α*_avg_), and first hyperpolarizability (*β*) of the PPAB chromophores. The UV-visible absorption spectra were analysed in solvents with different polarities, namely acetonitrile, chloroform, and toluene. The absorption values are tabulated in Table 2. All the studied molecules exhibit considerable absorption with high oscillator strength within the purple light wavelength range. For example, PPAB-1, PPAB-2, and PPAB-3 exhibit maximal absorption peaks at wavelengths of 579 nm, 643 nm, and 611 nm, respectively, in acetonitrile. The emission from the lowest excited singlet stated (S_1_), also known as fluorescence emission, which is calculated for PPAB-1, 2, and 3 in acetonitrile, is observed at wavelengths of 678 nm, 738 nm, and 674 nm, respectively. The notably high Stokes shift also indicates the CT nature of the studied molecules.

**Table 2:** The one-photon absorption wavelength (nm) with oscillator strength (*f*) and dominant orbital transition(s) and fluorescence emission details, as obtained using TD-DFT calculations employing ω*B97XD functional and 6-31g(d) basis set in different solvents

Molecules	Solvent medium	*λ*_abs_ (S_0_ → S_1_) in nm	Dominant transition	*λ*_F_ (S_1_ → S_0_) in nm
PPAB-1 (*ω* = 0.1069)	Acetonitrile	616.84 (*f* = 0.91)	H → L (98.10%)	677.51 (*f* = 1.31)
	Chloroform	626.18 (*f* = 0.95)	H → L (98%)	642.41 (*f* = 1.31)
	Toluene	629.36 (*f* = 0.97)	H → L (97.96%)	613.78 (*f* = 1.29)
PPAB-2 (*ω* = 0.1050)	Acetonitrile	643.30 (*f* = 1.18)	H → L (78.2%), [H−1] → L (17.6%)	738.0 (*f* = 1.71)
	Chloroform	643.91 (*f* = 1.20)	H → L (76.8%), [H−1] → L (19.2%)	685.0 (*f* = 1.7)
	Toluene	642.10 (*f* = 1.20)	H → L (76.56%), [H−1] → L (19.69%)	645.75 (*f* = 1.68)
PPAB-3 (*ω* = 0.1086)	Acetonitrile	610.76 (*f* = 0.81)	H → L (93.9%), [H−1] → L (3.1%)	673.83 (*f* = 1.11)
	Chloroform	616.83 (*f* = 0.85)	H → L (93.51%), [H−1] → L (3.53%)	642.41 (*f* = 1.03)
	Toluene	619.92 (*f* = 0.87)	H → L (93.4%), [H−1] → L (3.7%)	619.93 (*f* = 0.88)

### 3.4. Frontier molecular orbital analysis

**Fig. 3:**
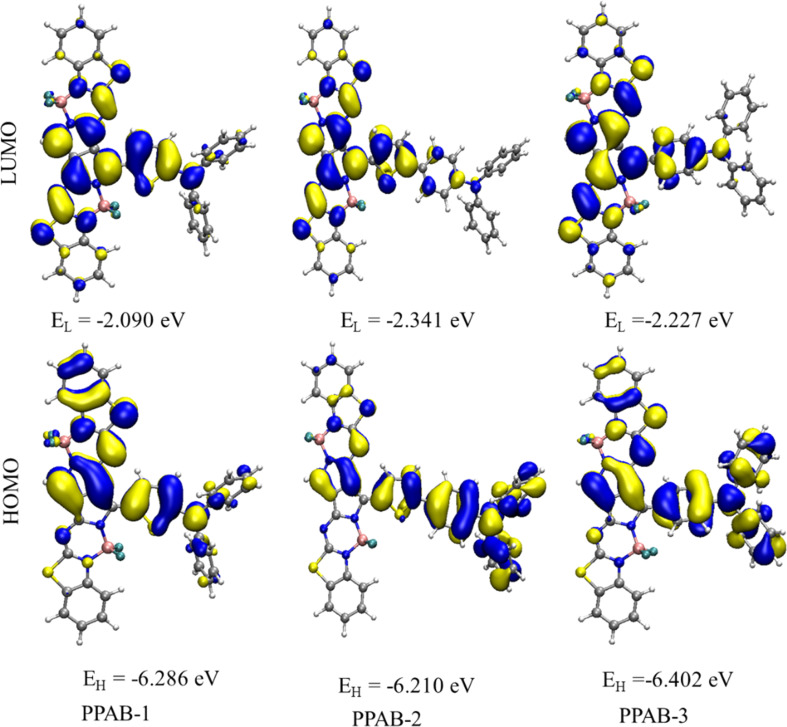
The HOMO and LUMO images with the energies of the D–π–A molecules, PPAB (1–3) in acetonitrile solvent.

To understand the nature of the studied chromophores, we have analysed the parameters, including the HOMO–LUMO energy gap and the percentage of CT and LE characters in the excited state. The assessment of the frontier molecular orbitals (FMOs) including, the HOMO–LUMO energy gap is crucial for understanding the structure–function relationships of molecules with their ICT nature. To explore the distribution patterns of molecular orbitals of HOMO and LUMO, we examined the FMO of the PPAB molecules in acetonitrile solvent using (IEF-PCM), as illustrated in Fig. 3. We also analysed the distribution patterns of the HOMO and LUMO (Fig. S1 and S2†) and energy gap of PPAB molecules in chloroform and toluene solvents using the (IEF-PCM), as shown in (Tables S4 and S5†). It is clear based on the FMO analysis in acetonitrile solvent that the HOMO densities of all PPAB molecules exhibit similarity as they are distributed over the donor moiety, indicating a π-bonding nature, and the LUMO densities are dependent upon the electron-withdrawing moieties, exhibiting a π-antibonding nature. The chemical stability, electrical, and optical characteristics of molecules are also reportedly well correlated using FMO analysis.^46^ Compounds with a lower HOMO–LUMO energy gap have higher chemical reactivity, and these compounds are highly polarizable, showing excellent NLO properties because of the higher possibility of ICT.^47^ The FMO analysis is done in three solvents with different polarities as well to study the effect of the polar solvent on the HOMO–LUMO energy gap. Furthermore, the energy gaps (Δ*E*_HL_) of the HOMO and the LUMO are shown in Table 3. The Δ*E*_HL_ value of PPAB-2 (3.869 eV) is lower than that of PPAB-1 and PPAB-3, thus the molecules possessing planarity and a low energy gap (Δ*E*) showed better NLO response. The molecule with a low energy gap has a high softness value and low hardness value. The ionization potential (IP), global electron affinity (EA), chemical hardness (*η*), chemical softness (*σ*) are calculated using the following equations.^48–50^

3 IP = −*E*_HOMO_

4 EA = −*E*_LUMO_

5
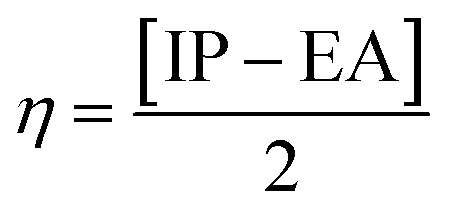
6
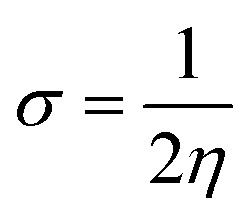


**Table 3:** The values of Δ*E*_HL_ (in eV) are obtained using ω*B97XD/6-31G(d) level of theory for the ground state PPAB molecules along with the CT and LE percentage. Global reactivity parameters of PPAB molecules are also reported, which are in Hartree

Molecules	HOMO (eV)	LUMO (eV)	Δ*E*_HL_ (eV)	CT (%)	LE (%)	in Hartree			
						IP	EA	*η*	*Σ*
PPAB-1	−6.286	−2.090	4.196	47.2	52.8	0.231	0.077	0.077	6.493
PPAB-2	−6.210	−2.341	3.869	56.9	43.1	0.228	0.086	0.071	7.042
PPAB-3	−6.402	−2.227	4.175	50.1	49.9	0.235	0.082	0.076	6.579

### 3.5. Electrostatic potential calculations

**Fig. 4:**
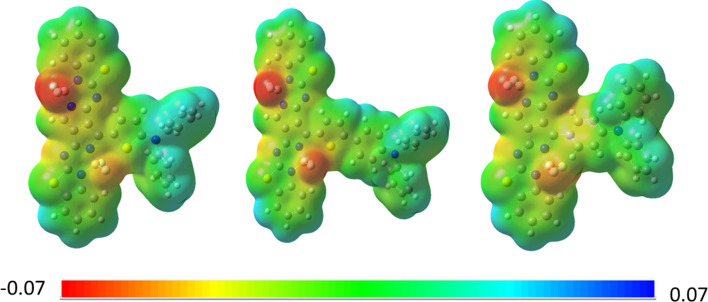
The electrostatic potential (ESP) of D–π–A molecules. The potential distribution in the ESP map increase in the order red < yellow < green < blue. The range of ESP map is ranging from −0.07 a.u. to 0.07 a.u.

The study of the electrostatic potential (ESP) provides a perceptive understanding of the distribution of charges within the molecule (Fig. 4). The ESP of aza-BODIPY molecules containing DPA and TPA groups exhibits significant differences. The region with a more negative potential, denoted by red regions, is predominantly situated on the F atoms of the aza-BODIPY group. However, the DPA and TPA groups exhibit a net positive potential, as indicated by the blue regions. This observation suggests that the aza-BODIPY group acts as the electron-acceptor group, while the DPA and TPA groups serve as the electron-donor group which results in ICT which play a major role in NLO properties of the molecule.

### 3.6. Dipole moment (*μ*), average polarizability (*α*_av_) and first-order hyperpolarizability (*β*)

The Dipole moment, average polarizability, and first hyperpolarizability of PPAB (1–3) are computed in the acetonitrile, chloroform and toluene are reported in Table 4. The important factors that affect the NLO properties of these molecules are also discussed.^51^ Higher values of *μ* indicate a higher polarity of the molecule in the ground state. The first hyperpolarizability (*β*) is a second order NLO property that can be tuned by using strong electron donor and acceptor groups, HOMO–LUMO energy gaps, and a high difference of *μ* between the ground and excited states. In general, the length of the CT of a molecule plays a major role in obtaining high *β* values. The *μ* values of PPAB-1, PPAB-2, and PPAB-3 are higher in the more polar solvent acetonitrile, with values of 13.834 *D*, 9.06 *D* and 7.32 *D*, respectively. This indicates that PPAB-1 consists of a low-lying excited state with stronger CT compared to PPAB-2 and PPAB-3. *p*-Nitroaniline is a well-known D–π–A model molecule that has gained a lot of attention from both experimentalists and theorists.^52^ The NLO properties of *p*-nitroaniline are also calculated using ω*B97XD/6-31G(d) functional and basis set to compare their results with PPAB (D−π−A) structures (see Table 4). The components of linear polarizability appear in the Gaussiaon16 output as follows: *α*_*xx*_, *α*_*xy*_, *α*_*yy*_, *α*_*xz*_, *α*_*yz*_ and *α*_*zz*_. The average polarizability (*α*) is a second rank tensor, which is calculated by the following eqn (7).7
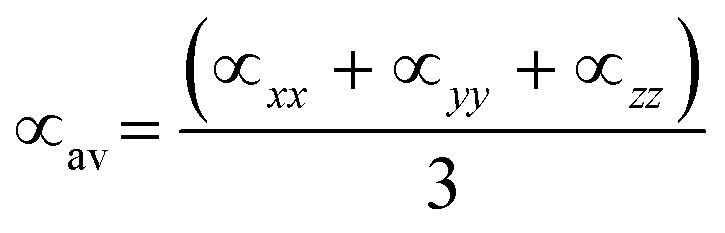


**Table 4:** The values of dipole moment (*μ*), average polarizability (*α*_av_) and *β*_total_ for molecules PPAB (1–3) in acetonitrile, chloroform, toluene, as obtained using DFT calculations employing ω*B97XD functional and 6-31g(d) basis set. The same properties computed for the reference molecule *p*-nitroaniline in chloroform is reported for comparison

Molecules	Solvent	*μ* (in debye)	*α*_av_ (in a.u.)	*β*_tot_ (in a.u.)	*β*_vec_ (in a.u.)	*β*_vec_/*β*_tot_	*β*_HRS_ at 1064 nm (in a.u.)
PPAB-1	Acetonitrile	13.834	975.29	30 756.93	−2373.01	−0.08	62 071.27
	Chloroform	12.22	893.68	22 857.57	−5559.25	−0.24	
	Toluene	10.90	824.96	17 492.15	−6582.11	−0.38	
PPAB-2	Acetonitrile	9.06	1168.80	144 576.99	−141783.9	−0.98	625 365.6
	Chloroform	8.352	1060.43	105 406.29	−103300.95	−0.98	
	Toluene	7.74	973.75	79 374.30	−77810.69	−0.98	
PPAB-3	Acetonitrile	7.32	973.71	58 277.21	−54095.37	−0.93	145 843.7
	Chloroform	6.76	889.35	45 283.05	−42349.34	−0.93	
	Toluene	6.25	600.38	35 303.46	−33245.92	−0.94	
*p*-nitroaniline	Chloroform	8.25	97.88	2211.76	−2190.31	−0.99	—

The static first order hyperpolarizability, (*β*_tot_) is a third rank tensor^53^ with twenty seven components, which can be reduced to ten components by virtue of Kleinman symmetry.^54^ Those ten components are *β*_*xxx*_, *β*_*xxy*_, *β*_*xyy*_, *β*_*yyy*_, *β*_*xxz*_, *β*_*xyz*_, *β*_*yyz*_, *β*_*xzz*_, *β*_*yzz*_ and *β*_*zzz*_ of the (3 × 3 × 3) tensor. These components have been used to define *β*_vector_ which is projected in the direction of the dipole moment of the molecule, as well as the *β*_tot_ which is computed by using the eqn (8).8
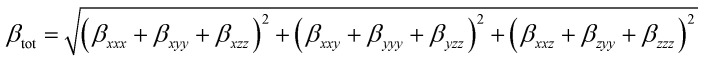


The *β*_tot_ and vector component of first hyperpolarizability (*β*_vec_) of molecules are obtained by using the tuned functional. The *β*_vec_ at the static limit is calculated using the eqn (9).9
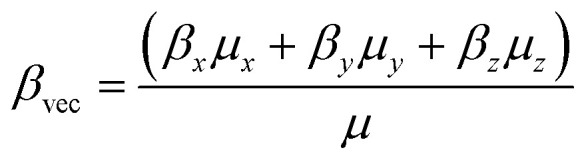


The ratio of *β*_vec_ and *β*_total_ is known to provide crucial information about the direction of charge transfer in an ICT-based molecule.^55^ Unidirectional charge transfer, indicated by the ratio of *β*_vec_ and *β*_total_ of unity, could be used to enhance the NLO response of a molecule.

Among the molecules studied, PPAB-2 showed the highest *β*_tot_ value, while PPAB-1 showed the lowest *β*_tot_ value. The higher *β*_tot_ of PPAB-3 than PPAB-1 could be assigned to the unidirectional charge transfer in the former, as indicated by the ratio of *β*_tot_ to *β*_vec_ close to unity. The *β*_tot_ of the three molecules is investigated in different solvents, and the results clearly indicate that the inclusion of the solvation effect leads to a substantial increase in the *β*_tot_ values of the compounds under investigation. As the extent of ICT is known to enhance with increasing solvent polarity, we can infer that ICT plays a significant role in shaping the NLO response of the PPAB (1–3).^56^ The *β*_tot_ value of the molecules in the acetonitrile solvent is much higher compared to the values observed in the less polar solvents. This finding suggests that the presence of a polarizable environment can effectively modulate the NLO response. The increase in *β* by modifying the dielectric environment around the chromophore is a potential method to greatly improve the performance of nonlinear optical systems. The components of *μ* as well as that of *β* are provided in Table 5. The results indicate that unlike PPAB-1, the hyperpolarizability of PPAB-3 is dominated by only one component (*β*_*y*_), indicating unidirectional charge transfer. Therefore, these results indicate that the *β* values of the PPAB molecules can be tuned using suitable donor and acceptor groups that facilitates unidirectional charge transfer.

**Table 5:** The components of dipole moment (*μ*) and first hyperpolarizability (*β*) of molecules PPAB (1–3), obtained using DFT calculations employing ω*B97XD functional and 6-31g(d) basis set. The same properties computed for the reference molecule *p*-nitroaniline in chloroform is reported for comparison

Molecules	in static						
	Medium	*μ*_*x*_	*μ*_*y*_	*μ*_*z*_	*β*_*x*_	*β*_*y*_	*β*_*z*_
PPAB-1	Acetonitrile	−3.84	3.85	0.01	−20038.21	−23331.91	−283.63
	Chloroform	3.38	3.42	−0.02	11 863.26	−19535.74	293.99
	Toluene	−2.99	3.07	0.04	−7023.23	−16017.20	−314.09
PPAB-2	Acetonitrile	3.55	−0.37	−0.06	−138144.82	42 625.39	−1261.99
	Chloroform	3.27	−0.33	0.005	−100709.30	31 093.47	−1149.007
	Toluene	3.03	−0.31	0.03	−75820.65	23 465.11	−946.91
PPAB-3	Acetonitrile	0.87	2.74	−0.08	4222.91	−58120.53	635.45
	Chloroform	0.87	2.51	0.08	1344.11	−45260.33	501.31
	Toluene	0.79	2.33	−0.07	600.38	−35296.31	379.53
*p*-Nitroaniline	Chloroform	0.37	−3.23	0.00	56.39	2211.04	2.43

### 3.7. Dynamic first-order hyperpolarizability

Hyper–Rayleigh scattering (HRS), or second harmonic Rayleigh scattering (SHLS) is a phenomenon in which light is scattered at the harmonic frequencies of the incident light.^57^ It is an incoherent, nonlinear optical process. It is important to take into account the effects of frequency dispersion correction in theoretical calculations because the hyperpolarizability obtained experimentally is in dynamic settings.^58^ To make our results useful for experimentalists and theoreticians alike, the frequency dependent NLO response in terms of the *β*_HRS_*β* (−2*ω*,*ω*,*ω*) is calculated.^59^ The *β*_HRS_ is an alternative to electric-field-induced second harmonic generation (EFISHG) for directly measuring the *β* value of all molecules, regardless of their symmetry or charge. The efficiency of SHG for a molecule is primarily determined by the molecular second-order nonlinear polarizability, or first hyperpolarizability, *β*_tot_. Castet *et al.* devised a method to assess the HRS response *β*_HRS_ (−2*ω*,*ω*,*ω*) effectively.^60^

The dynamic values used in this study were derived using an incident infrared wavelength (*λ*) of 1064 nm, which is typical of the Nd:YAG laser.^61^ Second-harmonic hyperpolarizability *β* (−2*ω*,*ω*,*ω*) can be measured in solution using Hyper–Rayleigh scattering, where *ω* is the frequency of the light field.^62,63^ The *β*_HRS_ hyperpolarizabilities of the molecules are calculated in acetonitrile. The second-order NLO susceptibility *X*_*lmn*_ is a third-rank tensor, and the presence of inversion symmetry in the molecule will make *X*_*lmn*_ = 0. In this context, molecules with inversion symmetry are not SHG active. The *β*_HRS_ (−2*ω*,*ω*,*ω*) can be described by eqn (10). The computed dynamic *β*_HRS_ (a.u.) values at the ω*B97XD/6-31g(d) level using 1064 nm incident wavelength in acetonitrile are reported in Table 4.10
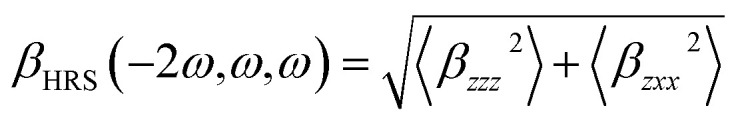


The *β*_HRS_ (−2*ω*,*ω*,*ω*) is the sum of 〈*β*_*zzz*_^2^〉 and 〈*β*_*zxx*_^2^〉 which are the average orientation of the molecular *β* tensor components. Where the *x*, *y*, and *z* are the axes of the molecular frame.

The values of the 〈*β*_*zzz*_^2^〉 and 〈*β*_*zxx*_^2^〉 tensors are calculated by the below eqn (11) and (12).11
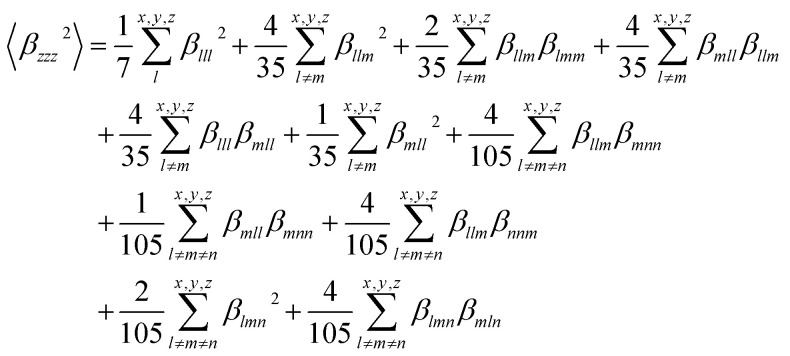
12
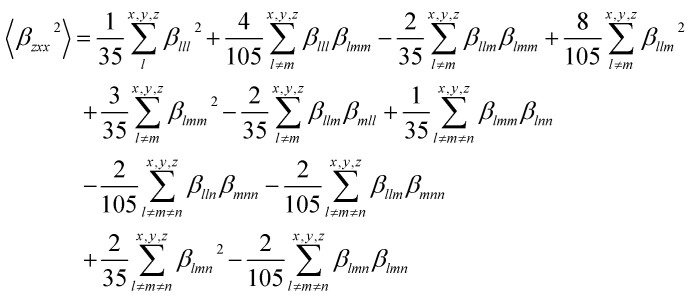


From the analysis of charge density difference, we observed that the amount of charge transfer is more for PPAB-2 which well collaborate with the high value of *β*_HRS_ for PPAB-2 (see Fig. S4†).

### 3.8. Two-photon absorption (2PA) calculations

2PA is a third-order nonlinear process, the simultaneous absorption of two photons of similar energy is called as two photon absorption.^64^ The increase in the percentage of CT nature in a molecule has also been reported to increase the 2PA cross-section. The 2PA cross-section is typically determined using the Goppert Mayer (GM) unit. The relationship between the GM and atomic unit is determined by the following eqn (13).^65^13
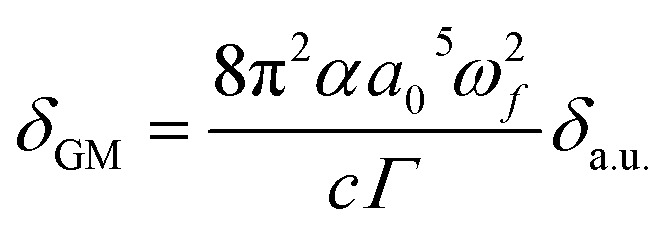


The variables in the eqn (13) are defined as follows: *α* represents the fine structure constant, *a*_0_ represents the Bohr radius; *c* represents the speed of light in a vacuum. The 2PA cross sections obtained for these molecules are large, which suggests that the investigated chromophores are promising as new organic materials for NLO. The 2PA cross section obtained for PPAB-2 (*δ*_TP_ = 6354 a.u. at 1771 nm) is five times higher than PPAB-3 (*δ*_TP_ = 1136 a.u. at 1560 nm, see Table 6). The two molecules consist of very similar molecular structures but two different lateral donor groups: TPA and DPA. The molecule's Two Photon absorption strength can be calculated by assessing the *δ*_TP_ value. However, this value alone does not offer a comprehensive understanding of the underlying physical process. To gain more insight, it is necessary to analyse the S tensor elements, which reveal the directionality of charge transfer and identify the specific tensor component that plays a significant role in the 2PA process. This information can be found in Table 6. The PPAB-1 molecule is situated within the *xy*-plane as a result, the highest value of the *S*_*xx*_ tensor element indicates that the transfer of charge will occur along the *x*-axis direction. The second and third largest components of the S tensor in PPAB-1 are *S*_*xy*_ and *S*_*yy*_ respectively. Similarly, in molecules PPAB-2 and PPAB-3 the *S*_*xx*_, *S*_*xy*_, and *S*_*yy*_ components make a substantial contribution. The component *S*_*xz*_ also plays a substantial role, suggesting that it has a three-dimensional charge-transfer nature. The findings show that PPAB-2 has a thienyl π linker while PPAB-3 has a phenylene linker, resulting in increased ICT and therefore higher two-photon absorption in molecule PPAB-2. Similarly, the more difference in charge density difference (Fig. S4†) and high strength of 2PA cross-section for PPAB-2 (see Table 6). For PPAB-1, the strength of 2PA cross-section and difference in charge density difference is comparative less.

**Table 6:** The two-photon tensor elements of D–π–A framework containing PPAB molecules were computed at the B3LYP/6-31G(d) level of theory in acetonitrile solvent phase

Molecules	2PA	*S*_*xx*_	*S*_*yy*_	*S*_*zz*_	*S*_*xy*_	*S*_*xz*_	*S*_*yz*_	*δ*_TP_ (a.u.)
PPAB-1	1158 nm	182.4	14.1	1.2	−56.4	−3.8	0.4	50.64
	1.07 eV							
PPAB-2	1771 nm	−3360.8	118.5	0.2	708.4	−23.4	−10.4	6354.24
	0.7 eV							
PPAB-3	1560 nm	368.0	−1337.0	2.2	−117.1	−2.7	6.1	1136.17
	0.79 eV							

## 4. Conclusion

This study presents a quantum chemical investigation of a series of novel ICT-based chromophores with a D–π–A configuration consisting of aza-BODIPY framework based pyrrolopyrrole aza-BODIPY (PPAB) as the electron acceptor group, and TPA or DPA acting as the electron donor group. The first hyperpolarizability of the studied molecules is found to be strongly dependent on the extent of the charge transfer between the electronic donor and the acceptor group through the π-electron bridge. The effect of functional and basis set on the absorption properties of the D–π–A–π–D molecules is studied to determine the best suitable functional/basis set combination for studies of NLO response. The first hyperpolarizability of D–π–A molecules is significantly higher than the reference molecule *p*-nitroaniline, indicating the suitability of these molecules for further exploration for designing NLO materials. The *β*_HRS_ of the ICT-based molecules designed in the present study is also calculated at 1064 nm in acetonitrile. Our results indicate that NLO response of novel ICT chromophores could be optimized through rational design by using suitable donor, acceptor and π-linkers that give rise to unidirectional charge transfer. The 2PA cross-section can also be significantly enhanced through careful modification of the donor and the linker substituents. These results indicate that the present study could serve as the basis for studies of second and third-order NLO properties of ICT-based chromophores consisting of PPAB electronic acceptors with suitable electronic donors and π-linkers with a D–π–A framework for future technological applications.

## Conflicts of interest

The authors declare no competing financial interest.

## Supplementary Material

RA-014-D4RA02861A-s001
