# Supplementary material for: Rational design and investigation of nonlinear optical response properties of pyrrolopyrrole aza-BODIPY-based novel push–pull chromophores
Source: RSC Adv. 2024 May 16;14(22):15560–70. doi: 10.1039/d4ra02861a (PMC11097754; doi:10.1039/d4ra02861a)
Supplement: RA-014-D4RA02861A-s001 [file RA-014-D4RA02861A-s001.pdf]

Electronic Supplementary Material (ESI) for RSC Advances.
This journal is © The Royal Society of Chemistry 2024

Supporting Information for

Rational Design and Investigation of Nonlinear Optical Response Properties of Pyrrolopyrrole Aza-BODIPY-Based Novel Push-Pull Chromophores

Naga Pranava Sree Kothoori[1], Pandiyan Sivasakthi[1,3], Mallesham Baithy[1], Ramprasad Misra[2,*], and Pralok K. Samanta[1,3,*]

[1]Department of Chemistry, School of Science, Gandhi Institute of Technology and Management (GITAM), Hyderabad-502329, India

[2]Institute for Biology, Experimental Biophysics, Humboldt-Universität zu Berlin, Berlin-10115, Germany

[3]Department of Chemistry, Birla Institute of Technology and Science Pilani (BITS Pilani), Hyderabad Campus, Hyderabad-500078, India

*Corresponding authors: ramprasad.misra@hu-berlin.de (RM); pralokkumar.samanta@hyderabad.bits-pilani.ac.in (PKS)

Table S1: Absorption data comparison of Molecules (1-3) using different functionals. The experimental values of the absorption maxima are obtained from Ref.1. All values are in eV. The tuned ω values are also mentioned.

Molecules	[1]Expt.	ω*B97XD	ωB97XD	CAM-B3LYP	B3LYP	PBE1PBE
Mol-1	1.52	1.65 (ω=0.0938)	1.80	1.77	1.57	1.61
Mol-2	1.56	1.7 (ω=0.0901)	1.90	1.85	1.45	1.53
Mol-3	1.70	1.79 (ω=0.0961)	2.03	1.98	1.60	1.67

Table S2: Values of μ, α_{av}, β_{total} and β_{vec} of molecules (1-3) obtained using the ω*B97XD tuned functional and 6-31g(d) basis set

Molecule	μ (in Debye)	α_{av} (in a.u.)	β_{tot} (in a.u.)	β_{vec} (in a.u.)	$\beta_{vec/btot}$
Mol-1	0.00077	1359.01433	8.76496	7.74446	0.88357
Mol-2	0.00385	1627.14600	76.61045	-74.29291	-0.96975
Mol-3	0.00137	1258.85700	5.26522	-2.61715	-0.49706

Table S3: The x, y and z components of dipole moment and hyperpolarizability of molecules (1-3) as obtained using the ω*B97XD tuned functional and 6-31g(d) basis set

Molecule	μ_x	μ_y	μ_z	β_x	β_y	β_z
Mol-1	-0.00029	-0.00005	-0.00008	-8.54920	1.90096	0.34938
Mol-2	0.0014	-0.00044	0.00014	-74.30075	6.34580	-17.55820
Mol-3	0.00002	-0.00040	0.00036	4.28012	1.27342	-2.78954

Table S4: HOMO, LUMO and energy gap (ΔE_{HL}) of PPAB (1-3) molecules in eV in chloroform.

Molecules	HOMO	LUMO	ΔE_{HL}
PPAB-1	-6.208	-2.028	4.180
PPAB-2	-6.178	-2.273	3.905
PPAB-3	-6.341	-2.165	4.176

Table S5: HOMO, LUMO and energy gap (ΔE_{HL}) of PPAB molecules in eV in toluene.

Molecules	HOMO	LUMO	ΔE_{HL}
PPAB-1	-6.151	-1.979	4.172
PPAB-2	-6.156	-2.218	3.938
PPAB-3	-6.299	-2.113	4.186

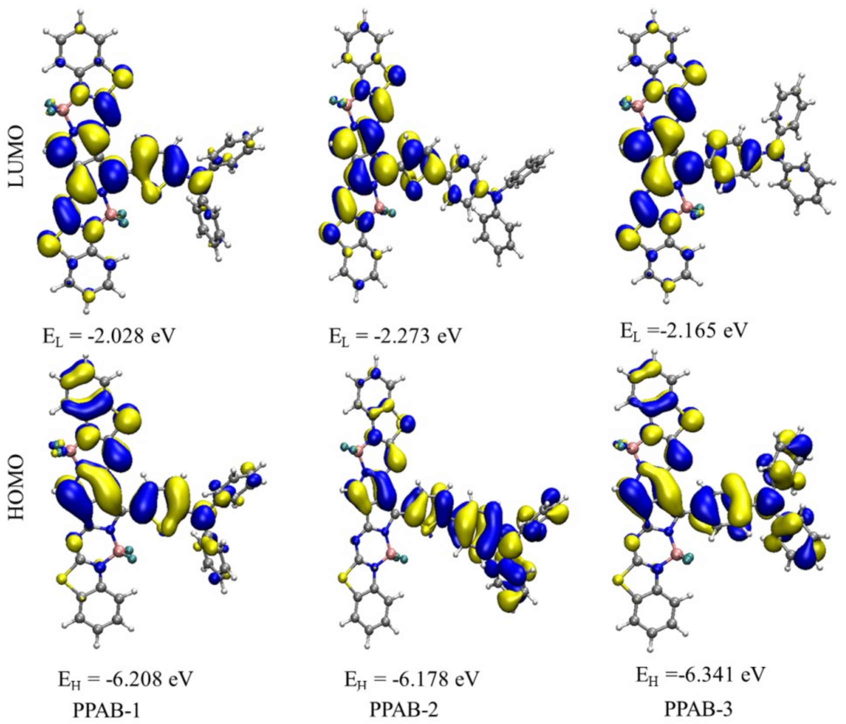

Fig. S1 The HOMO and LUMO picture of PPAB (1-3) in chloroform with their energies.

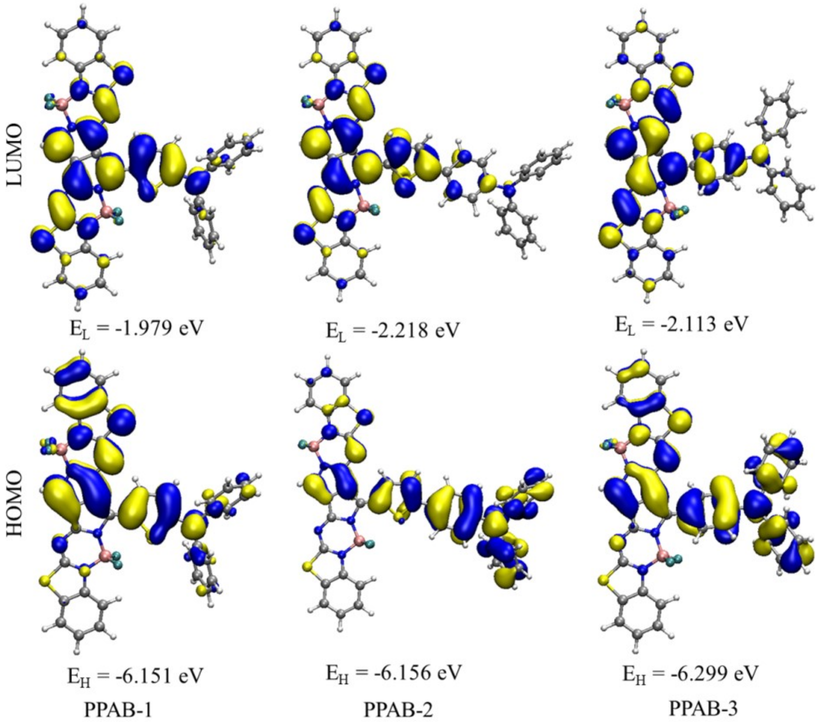

Fig. S2 The HOMO and LUMO picture of PPAB (1-3) in toluene with their energies.

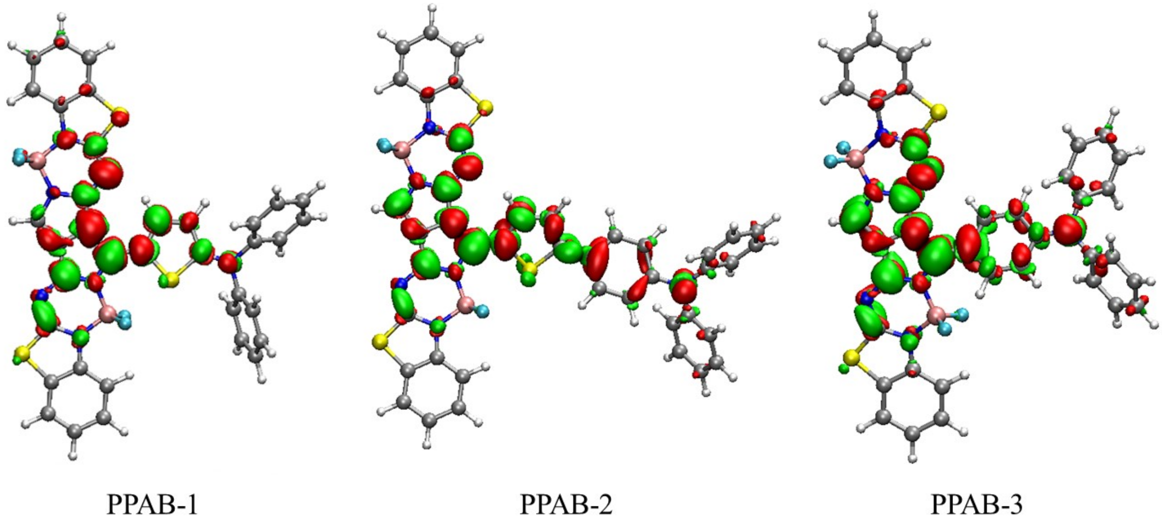

Fig. S3 The Electron density for PPAB molecules in acetonitrile. (Isovalue=0.001, +ve = green, -ve = red).

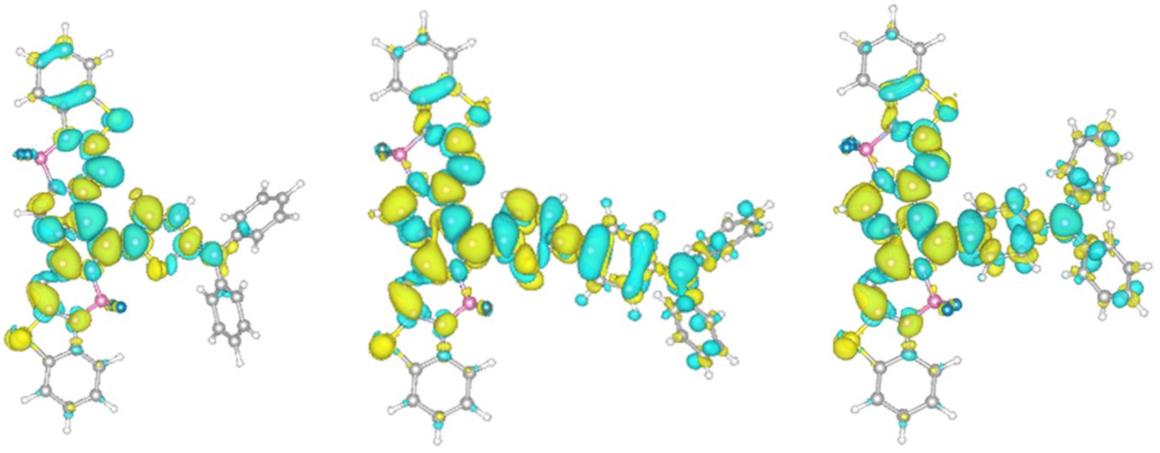

PPAB-1 PPAB-2 PPAB-3

Fig. S4 The charge density difference of PPAB molecules in acetonitrile. (Isovalue=0.0005, +ve = yellow, -ve = blue).

Reference

1 Y. Wang, S. Mori, H. Nakanotani, C. Adachi and S. Shimizu, Post-Modification of Pyrrolopyrrole Aza-BODIPY toward High Near-Infrared Fluorescence Brightness, *Org. Lett.*, 2023, **25**, 3040–3044.
